# Supplementary material for: Scaling and data collapse from local moments in frustrated disordered quantum spin systems
Source: Nat Commun. 2018 Oct 22;9:4367. doi: 10.1038/s41467-018-06800-2 (PMC6197223; doi:10.1038/s41467-018-06800-2)
Supplement: Supplementary file 1 — Supplementary Information [file 41467_2018_6800_MOESM1_ESM.pdf]

Scaling and data collapse from local moments in frustrated disordered quantum spin systems

SUPPLEMENTARY INFORMATION

This Supplementary contains important details for the calculations described in the main text. It is structured as follows. We begin by considering the effect of DM interactions on modifying the two-spin singlet state, as well as the competition of these effects with an applied magnetic field. We then consider the RG flow under strong disorder RG, as appropriate for random singlets, in the presence of DM interactions. Then we compute the expected scaling phenomenology and discuss the various cases for q. Finally we relate the scaling forms for various observables including heat capacity, NMR lifetimes and magnetization.

The starting point for the calculations in this Supplementary is a picture of emergent couplings with the topology of a random network, a distribution with a long power-law tail, and an associated strong-disorder RG flow. To understand the emergence of such self-similar power laws out of a microscopic lattice model with merely flat disorder distributions, we refer the interested reader to the RG flows discussed in Ref. 1. A picture for how the RG flow is initiated out of the UV lattice scale for one simple class of microscopic models is depicted in Fig. 1.

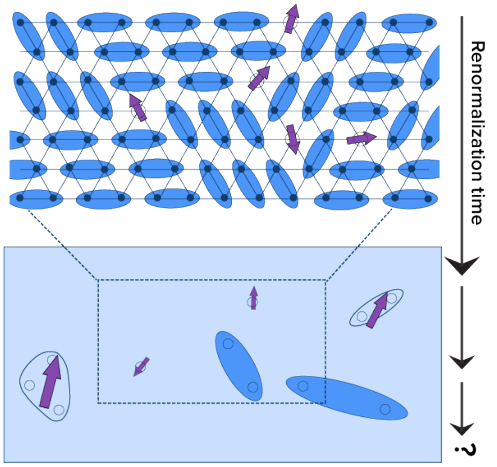

Supplementary Figure 1. **RG flow from a class of weakly-disordered lattice models into a random network with power law distributions.** Disordering short-ranged valence bonds is found, under certain conditions and even for weak randomness, to necessarily lead to defects that carry protected spin-1/2 moments. The resulting spin moments form a random network with power-law distributions, an appropriate setting for strong-disorder RG. Regardless of the ultimate fixed point of the RG, which in 2D and 3D is unknown, the system is described by emergent power laws across some low energy regime associated with the RG flow. Adapted from Ref. 1, CC-BY-3.0.

Supplementary Note 1: Two spins with DM interactions

To see the effects of DM interactions on the valence bond states, consider the following Hamiltonian for two spins i, j,

$$\mathcal{H}_{ij} = J(\vec{S}_i \cdot \vec{S}_j) + \vec{D} \cdot (\vec{S}_i \times \vec{S}_j) - \vec{H} \cdot (\vec{S}_i + \vec{S}_j) \qquad (1)$$

where in addition to a general DM term and magnetic field, for simplicity here the symmetric spin interactions are taken to consist of pure Heisenberg exchange; below we discuss generic SO(3) breaking exchanges. Defining the total spin operator $\vec{S}_{\rm tot} \equiv \vec{S}_i + \vec{S}_j$, the Hamiltonian up to an overall energy shift may be written as

$$\mathcal{H}_{ij} = \frac{J}{2}(\vec{S}_{\rm tot})^2 + \frac{|D|}{2}(T_D + T_D^\dagger) - \vec{H} \cdot \vec{S}_{\rm tot} \qquad (2)$$

where the DM term mixes the singlet with the member of the triplet manifold that has zero total spin moment along the DM vector,

$$T_D \equiv i \; |\text{singlet}\rangle\langle\text{triplet}; \; (\vec{S}_{\rm tot} \cdot \vec{D} = 0) \; | \qquad (3)$$

At zero field $H = 0$, the ground state is a mixture of the spin-singlet state and this $\vec{S}_{\rm tot} \cdot \vec{D} = 0$ member of the triplet manifold, set by the Hamiltonian matrix $2\mathcal{H}_{H=0} = J\sigma^3 + |D|\sigma^2$ acting on the basis of these two states. The ground state is nondegenerate; for small D/J, it is modified from the singlet state only perturbatively in D/J.

For larger fields near resonance $H \approx J$ it is useful to project the Hamiltonian to the low energy space spanned by the singlet and the member of the triplet with maximal total magnetization along the magnetic field direction. In this 2-level subspace, the Hamiltonian, again up to a constant, is

$$\mathcal{H}_{H\approx J} = \frac{1}{2}(|H| - J)\sigma^3 + \frac{1}{2\sqrt{2}}\left(D_1\sigma^2 - D_2\sigma^1\right) \qquad (4)$$

where σ are Pauli matrices acting on the (singlet, total-spin-up-triplet) basis, and D_1, D_2 are the components of the DM vector perpendicular to the magnetic field axis.

Indeed this equation, with modified parameters, describes the general scenario reached by adding generic spin-orbit-coupled interactions to the symmetric spin exchange matrix $J_{ij}^{\mu\nu} S_i^\mu S_j^\nu$. Without the DM term the singlet state remains an eigenstate and we shall assume the symmetric exchanges are sufficiently antiferromagnetic so that the singlet is the ground state of $J_{ij}^{\mu\nu} S_i^\mu S_j^\nu$. The triplet-manifold eigenstate brought down by the field is modified. The resulting Hamiltonian is described by Eq. 4 with J interpreted as an effective exchange parameter that may depend on the direction of $\vec{H}$, and D_1, D_2 interpreted as two particular components of the DM vector.

Supplementary Note 2: Strong-disorder RG step: integrating out two spins with DM interactions

The strong disorder RG (SDRG) step entails integrating out a pair of spins and considering the renormalization of interaction for every other remaining pair. We perform it analytically, within a controlled hierarchy of parameters, to establish a recursion condition that must be satisfied by any fixed-point distribution. The recursion condition ensures (1) that the Heisenberg SDRG is not modified by the presence of DM interactions, and (2) that the flow of DM interactions preserves the separation of scales discussed in the main text and used in the derivation of the specific heat scaling (Supplementary Note 3).

In one-dimensional systems SDRG is possible to perform analytically in certain cases[2]. 1D systems are special for a variety of reasons, most importantly here since integrating out two neighboring spins in a spin chain results in a system that is still exactly described as a spin chain. Numerical implementations of SDRG in higher dimensional systems, using various approximations, generally find that the SDRG assumptions become uncontrolled and the fate of the ultimate fixed point remains controversial[3–6].

Here we avoid such unresolved questions about the fixed point of $d > 1$ SDRG by restricting ourselves to a particular narrow question: how does the presence of DM interactions change the SDRG? We are able to answer this question rigorously by performing a single SDRG step analytically and noting two observations about the resulting recursion relation. First, we find that weak DM interaction does not enter the renormalization of the symmetric (e.g. Heisenberg) exchanges. The SDRG for Heisenberg exchange therefore proceeds identically as in the case without DM interaction. Second, we find that the renormalization of DM interactions preserves a parametric separation of scales between the DM and symmetric exchanges on each bond. The relative scale of the DM interactions therefore does not change and in particular also the control parameter for the first result is preserved under RG. In this precise sense, the DM interactions are merely spectators to the standard SDRG, which proceeds as usual.

Computation. To perform the single SDRG step it suffices to look at each cluster of four spins containing the strong pair. We assume weak DM interactions; this is the case for weak spin-orbit coupling as well as for strong spin-orbit coupling with some approximate microscopic or emergent inversion or mirror symmetries. As the starting point for an SDRG step we must postulate that the quantum state of a portion of the system, namely the fraction of spins that participate in the random-singlet-type regime, is described by a power law distribution of exchange energies. Computing the SDRG step in the presence of weak DM interactions, we find that in 1D the DM and Heisenberg terms scale identically, while in 2D they scale at the same order with some distinctions in detail. The resulting *random-singlet* state has each spin paired with another into a nondegenerate frozen state, that differs from the spin-singlet state by the addition of a spin quadrupole (nematic) moment that preserves time-reversal, as is anyway required by the lack of spin rotation symmetry.

Consider a pair (1,2) of strongly coupled spins, with Heisenberg exchange J_{12} as well as DM exchange D_{12}, and consider weak Heisenberg as well as DM interactions between spins 1,2 and two additional spins 3,4. The ground state of $H_{12} = J_{12}H_{12}^{\mathrm{Heis}} + D_{12}H_{12}^{\mathrm{DM}}$ is no longer a spin-singlet under spin rotations, but nevertheless is a unique state. The low energy manifold has spins 1,2 in the ground state of H_{12}, while the bare interactions H_{34}^0 gain a renormalized contribution by virtual excitations of the (1,2) excited states, as $H_{34}^0 \to H_{34}^0 + \Delta H_{34}$. Here and below we take J_{12} to be larger than all other Hamiltonian parameters i.e. we work within lowest order perturbation theory in $1/J_{12}$. Integrating out spins 1,2, we find the renormalized interaction ΔH_{34} which can be expressed with coefficients J_{34}, D_{34} as $\Delta H_{34} = J_{34}H_{34}^{\mathrm{Heis}} + D_{34}H_{34}^{\mathrm{DM}}$. In writing the results below we specialize to the case where all DM vectors share the same orientation. The resulting expressions are directly applicable to the $q = 1$ case, e.g. a 2D layered system with effective reflection symmetry across each 2D lattice plane. This is the relevant analysis for the experimental data shown in the figures of the main text, as well as for the data of Ref. 7.

The renormalized contributions to the Heisenberg and DM interactions are given by the following expressions. (We write the DM vector on an oriented bond (i, j) as a pseudoscalar D_{ij}.) The symmetric splitting remains exactly unchanged by the presence of DM interactions,

$$J_{34} = \frac{(J_{14} - J_{24})(J_{23} - J_{13})}{2J_{12}} \tag{5}$$

Only the emergent DM interaction is modified,

$$\begin{aligned} D_{34} = & \frac{D_{12}\,(J_{14}J_{23} - J_{13}J_{24})}{2J_{12}^2} \\ & - \frac{(D_{13} - D_{23})(J_{14} - J_{24})}{2J_{12}} \\ & - \frac{(D_{14} - D_{24})(J_{13} - J_{23})}{2J_{12}} \end{aligned} \tag{6}$$

Importantly, if we assume a parametric separation of scale between the DM interaction and the symmetric splitting on each bond, this small parameter appears in all three terms above, and thus the resulting DM interaction ratio D_{34}/J_{34} appears with precisely the same small parameter.

The conclusion arising from this result, which remains true for arbitrary orientations of DM interactions, is thus as follows. A distribution of symmetric (e.g. Heisenberg) and antisymmetric (DM) interactions, characterized by a parameter denoting the ratio of strengths of antisymmetric and symmetric interactions on every bond, shows an SDRG evolution that obeys two important properties.

1. The distribution of symmetric exchanges (J's) evolves in exactly the same way regardless of the presence or absence of DM interactions. The DM interactions are spectators and do not modify the SDRG evolution of Heisenberg exchanges.

2. The distribution of DM interactions evolves in a way that does depend on the distribution of J's but nevertheless preserves the condition of separation of scales, namely the ratio of DM and J energy scales is preserved. The overall scale of the distribution of $|D_{ij}/J_{ij}|$ remains fixed under SDRG.

This result allows us to take the known results on SDRG with Heisenberg terms and apply them directly to the present case with additional DM interactions.

Supplementary Note 3: Derivation of scaling in a magnetic field

Here we discuss in detail the impact of DM interactions on the scaling relation for the density of states, as observable by heat capacity, in a magnetic field.

The entropy distribution at low energies is set by the distribution of couplings that emerges under RG flow. Consider then the distribution of bonds picked by the strong-disorder RG. It defines the (highly correlated) distributions of three energy parameters: (1) the symmetric splitting J; (2) the antisymmetric splitting set by the components D_i of the DM vector $\vec{D}$; (3) the resulting true splitting between the lowest state and the second state. Let us denote the distributions of these parameters by P_1, P_2, P_3 respectively. The Hamiltonian matrix elements for the lowest two states are given above, and the resulting splittings are as follows. With no magnetic field, this splitting is

$$\Delta E[H=0] = \frac{1}{2}\sqrt{J^2 + |D|^2} \tag{7}$$

In a magnetic field, this splitting (see Eq. 4) becomes

$$\Delta E = \frac{1}{2\sqrt{2}}\sqrt{2(|H|-J)^2 + D_1^2 + D_2^2} \tag{8}$$

where D_1, D_2 are the components of the DM vector that lie perpendicular to the magnetic field $\vec{H}$. The entropy distribution seen directly by $C[T]$ at zero field is the distribution of the splittings $P_3[E]$. Self consistency within the strong-disorder RG framework requires a broad tail for $P_3[E]$, generally power law $P_3[E] = E^{-\gamma}$, with γ defined by the specific heat exponent at zero field,

$$C[H=0][T] \sim T^{1-\gamma}.$$

Now let us add a magnetic field and consider the scaling of specific heat at temperatures far below the Zeeman energy, $T \ll H$.

First recall the case of no DM interactions, $D = 0$. Here the field picks out the distribution $P_3[H]$, ie P_3 at energy $\Delta E = H$. Now we may linearize P_3 for energy splittings E near $\Delta E = H$. It has an analytic polynomial expansion, which to first order may be written as $P_3[E] = P_3[H] + c_1(E-H)$. The specific heat picks out all states with energy $\Delta E < T$. Due to the linearization, $C[H,T] \approx TP_3[H]$.

Now consider the case of small but nonzero DM interactions. Let us count the states with excitation energy $\Delta E < T$. J is set by the distribution $P_1[J-H] = P_1[H] + c_1(J-H)$. The conditional distribution for each component D_i of the DM vector $\vec{D}$, conditioned on being given a bond with a particular Heisenberg exchange $J \approx H$, may be taken to be approximately Gaussian with some width set by this $J \approx H$. To reach a resonance with $\Delta E < T$ in the regime where $T \ll H$, we must have $D_i \ll H$ and thus the distribution of D_i of interest here is uniform and may be approximated by its value at the origin, $P_2[D_i] \approx P_2[0]$. Since the width of the distribution P_2 is proportional to $J \approx H$, normalization sets its overall amplitude by a factor proportional to $1/J \approx 1/H$, giving $P_2[D_i] \approx P_2[0] = c_2/H$. The number of states with $\Delta E < T$ is then given by $TP_1[H] \sim T/H^\gamma$ times a factor of T/H for each relevant component D_i.

Supplementary Note 4: Scaling of specific heat in a magnetic field

Due to quantum mechanical level repulsion, a low excitation energy $\Delta E < T$ entails conditions on the amplitude of three parameters: $||H| - J| < T$, $|D_1| < T$, and $|D_2| < T$. We must now consider three distinct physical scenarios, depending on the crystal symmetries or the effective approximate symmetries that emerge in SDRG, as well as potentially depending on the direction of the magnetic field. Recall that the scaling relation is $C[H,T] \sim T^{1-\gamma}$ for $H \ll T$, and is

$$C[H,T] \sim \frac{T^{q+1}}{H^{q+\gamma}} \tag{9}$$

for $T \ll H$.

Case (1): $\vec{D} \times \vec{H} = 0$. This is the case if symmetries constrain the DM vector to lie along a particular axis, and the magnetic field is taken to lie along the same axis. Then the specific heat scaling reduces to the case with no DM terms, and we find $q = 0$.

Case (2): only one component of $\vec{D}$ perpendicular to $\vec{H}$ is nonzero. This is the case if symmetries constrain the DM vector to lie along a particular axis, for any magnetic field direction away from that special axis (or for a powder average). [1] For example, this can occur when there is a mirror symmetry consisting of reflection across

[1] Case (2) can also occur if symmetries constrain the DM vector to lie in a plane and the magnetic field is not oriented perpendicular to this plane.

the 2D plane: this symmetry constrains $\vec{D}$ to lie perpendicular to the plane. Even if the crystal does not have such a symmetry microscopically, it may be reasonable to expect that, within a 2D magnetic layer, the magnetic exchanges that develop under RG will depend only on the single layer, and that such an mirror symmetry may emerge as an approximate symmetry under RG. In particular, within an approximation where each magnetic layer is treated independently as a purely 2D system, a given SDRG step will involve only the configuration of valence bonds within the 2D layer that was generated in previous RG steps, and reflection across the plane becomes a symmetry. In this case, only one component D_i is relevant, and we find $q = 1$.

Case (3): both D_1, D_2 are nonzero. This is the case if no symmetries constrain the DM vector. (This is also the case if symmetries constrain the DM vector to lie in a plane, and the magnetic field is oriented perpendicular to this plane.) Then both D_1, D_2 are relevant, and we find $q = 2$.

Thus we find a different scaling form, with different powers of T/H, depending on the crystal or emergent symmetries as well as the direction of the magnetic field. The linear scaling of 1T-TaS_2 ($q = 0$) may be understood by noting that the inversion centers in the 1T-TaS_2 crystal structure forbid DM interactions from being generated not only for nearest-neighbor bonds but also for 2nd, 3rd, 4th, etc neighbors, so it may be reasonable that no sizable DM interactions arise during RG. Quadratic scaling ($q = 1$), as in $H_3LiIr_2O_6$ and $LiZn_2Mo_3O_8$, can arise for generic or powder-averaged field directions through an emergent approximate mirror symmetry consisting of reflections across the 2D layer, a reasonable scenario for layered magnets. Cubic scaling ($q = 2$) may thus be expected to arise in valence bond materials with 3D lattices, such as Ba_2YMoO_6[8]. Experimental observation of the $q = 2$ case is left for future work.

Supplementary Note 5: Magnetization scaling and NMR $1/T_1$

It is instructive to contrast the heat capacity with other experimental quantities. Magnetization, in particular, does not access the same information on the entropy of the spectrum. Magnetization and susceptibility scaling has been previously discussed for SO(3) random singlets in the context of doped semiconductors [9–11], as well as theoretically[12] in the context of observed susceptibility scaling from the dilute impurity spins of synthetic herbertsmithite[13]; the scaling is $M[H,T] \sim H^{1-\gamma}$ for $T \ll H$ and $M[H,T] \sim HT^{-\gamma}$ for $H \ll T$. [2] Note that magnetization at $T \ll H$ is determined by counting all states with $J < H$: any information on the low energy states with $\Delta E < T$, which determine the specific heat scaling $C[H,T]$, is not captured by the magnetization or susceptibility at $T \ll H$. In particular the presence of weak DM interactions does not modify these expressions for magnetization or susceptibility scaling.

Interestingly however, Maxwell's relation between entropy and magnetization gives an additional subdominant correction to the magnetization scaling at $T \ll H$; this correction encapsulates the heat capacity and does involve the q exponent. Observe that the Maxwell relation $\partial S/\partial H = \partial M/\partial T$ (required by a thermodynamic potential $G[H,T]$ with curl[grad[$G[H,T]$]] = 0) conveys information about the entropy to the temperature derivative of magnetization. At $T \ll H$ the conventional magnetization scaling is expressed as $M \sim H^{1-\gamma}$ giving $\partial M/\partial T = 0$. Maxwell's relation is satisfied by adding a subdominant negative term to the $T \ll H$ magnetization scaling, yielding the scaling function

$$\frac{M[H,T]}{H} \sim \frac{1}{H^\gamma} F_q^M\left[\frac{T}{H}\right] \tag{10}$$
$$F_q^M[X] \sim \begin{cases} 1 - m_1 X^{q+2} & X \ll 1 \\ X^{-\gamma} & X \gg 1 \end{cases}$$

Since we are in the $T \ll H$ regime, this additional $(T/H)^{q+2}$ correction is quite small. This subdominant magnetization scaling coefficient is given by $m_1 = (q+\gamma)/((q+1)(q+2))((C/M)\,(H/T)^{q+1})[T{=}0]$ when heat capacity $C \sim H^{-q-\gamma}T^{q+1}$. This relation between the entropy measured in $C[H,T]$ and the magnetization $M[H,T]$ is also visible in the experimental data of Ref. 7. There magnetization was found to obey a scaling relation that appears to be roughly captured by $M \sim HT^{-1/2}$ for $H \ll T$, and, for $T \ll H$, $M \sim H^{1/2}$ with an additional subdominant correction consistent with a $(T/H)^3$ term added with a negative sign. Taking $\gamma \approx 1/2$ as extracted independently from the scaling of specific heat at zero field, these measurements are consistent with the theoretical expectations.

The scaling of heat capacity, equivalent to the scaling of the entropy, is also closely related to the scaling of $1/T_1T$ where T_1 is the NMR spin-lattice relaxation time. The quantity $(T_1T)^{-1}$ is related to the momentum-averaged (local) imaginary part of the spin susceptibility. Thus it serves as a measure of the spin-carrying states in the spectrum. NMR $1/T_1$ measurements complement the heat capacity by providing the information that the gapless spectrum carries spin excitations — nontrivial information for weak SOC, which is of course satisfied by the spectrum associated with breaking valence bonds.

Note however that the broad distribution of couplings inherently implies that the spin-lattice relaxation, measured by the NMR lifetime T_1, should be described by a highly stretched exponential. A fit to an exponential with a single lifetime T_1 will likely not be appropriate. (When considering a single lifetime T_1, it is worth noting that the NMR Korringa law is a fine tuned case appropriate only for a metal: more generally the $1/T_1$ is linear

[2] Large spin clusters which may arise in the course of the RG flow can contribute additional Curie (free spin) terms to the magnetization and susceptibility scaling.

in the density of states, $1/T_1 \sim C/\Gamma$ with some lifetime Γ which is a priori not related to the density of states, and not known.) In general there may be two contributions to the spin-lattice relaxation: one from the minority of spins that participate in the random-singlet scaling, and another from the phase of the bulk of the sites. However generally the fluctuations will be dominated by the few sites in the tail of the energy distribution, i.e. the minority of spins in the random-singlet regime, producing a continuum of lifetimes for the spin-lattice relaxation and rendering exponential decay fits with a single lifetime T_1 difficult to interpret.

[1] Itamar Kimchi, Adam Nahum, and T. Senthil, "Valence bonds in random quantum magnets: Theory and application to $YbMgGaO_4$," Phys. Rev. X **8**, 031028 (2018).
[2] Daniel S. Fisher, "Random antiferromagnetic quantum spin chains," Phys. Rev. B **50**, 3799–3821 (1994).
[3] R. N. Bhatt and P. A. Lee, "Scaling studies of highly disordered spin-1/2 antiferromagnetic systems," Phys. Rev. Lett. **48**, 344–347 (1982).
[4] Olexei Motrunich, Siun-Chuon Mau, David A. Huse, and Daniel S. Fisher, "Infinite-randomness quantum ising critical fixed points," Phys. Rev. B **61**, 1160–1172 (2000).
[5] Y.-C. Lin, R. Mélin, H. Rieger, and F. Iglói, "Low-energy fixed points of random heisenberg models," Phys. Rev. B **68**, 024424 (2003).
[6] C. R. Laumann, D. A. Huse, A. W. W. Ludwig, G. Refael, S. Trebst, and M. Troyer, "Strong-disorder renormalization for interacting non-abelian anyon systems in two dimensions," Phys. Rev. B **85**, 224201 (2012).
[7] K. Kitagawa, T. Takayama, Y. Matsumoto, A. Kato, R. Takano, Y. Kishimoto, S. Bette, R. Dinnebier, G. Jackeli, and H. Takagi, "A spin-orbital-entangled quantum liquid on a honeycomb lattice," Nature **554**, 341 (2018).
[8] M. A. de Vries, A. C. Mclaughlin, and J.-W. G. Bos, "Valence bond glass on an fcc lattice in the double perovskite Ba_2YMoO_6," Phys. Rev. Lett. **104**, 177202 (2010).
[9] R N Bhatt, "Magnetic properties of doped semiconductors," Phys. Scr. **1986**, 7 (1986).
[10] M. P. Sarachik, A. Roy, M. Turner, M. Levy, D. He, L. L. Isaacs, and R. N. Bhatt, "Scaling behavior of the magnetization of insulating Si:P," Phys. Rev. B **34**, 387–390 (1986).
[11] A. Roy, M.P. Sarachik, and R.N. Bhatt, "Scaling behavior of the magnetization of Si:B," Solid State Commun. **60**, 513 – 516 (1986).
[12] R. R. P. Singh, "Valence bond glass phase in dilute kagome antiferromagnets," Phys. Rev. Lett. **104**, 177203 (2010).
[13] J. S. Helton, K. Matan, M. P. Shores, E. A. Nytko, B. M. Bartlett, Y. Qiu, D. G. Nocera, and Y. S. Lee, "Dynamic scaling in the susceptibility of the spin-$\frac{1}{2}$ kagome lattice antiferromagnet herbertsmithite," Phys. Rev. Lett. **104**, 147201 (2010).
